# Bioinformatics Analyses of the Role of Vascular Endothelial Growth Factor in Patients with Non-Small Cell Lung Cancer

**DOI:** 10.1371/journal.pone.0139285

**Published:** 2015-09-30

**Authors:** Ying Wang, Lu Huang, Shuqiang Wu, Yongshi Jia, Yunmei Yang, Limin Luo, Aihong Bi, Min Fang

**Affiliations:** 1 Department of Radiation Oncology, Zhejiang Provincial People's Hospital, Hangzhou, 310014, China; 2 Department of Orthopedics, the Second Affiliated Hospital, School of Medicine, Zhejiang University, Hangzhou, 310009, China; 3 Department of Geriatrics, the First Affiliated Hospital, School of Medicine, Zhejiang University, Hangzhou, 310003, China; Public Health Research Institute at RBHS, UNITED STATES

## Abstract

**Purpose:**

This study was aimed to identify the expression pattern of vascular endothelial growth factor (VEGF) in non-small cell lung cancer (NSCLC) and to explore its potential correlation with the progression of NSCLC.

**Methods:**

Gene expression profile GSE39345 was downloaded from the Gene Expression Omnibus database. Twenty healthy controls and 32 NSCLC samples before chemotherapy were analyzed to identify the differentially expressed genes (DEGs). Then pathway enrichment analysis of the DEGs was performed and protein-protein interaction networks were constructed. Particularly, VEGF genes and the VEGF signaling pathway were analyzed. The sub-network was constructed followed by functional enrichment analysis.

**Results:**

Total 1666 up-regulated and 1542 down-regulated DEGs were identified. The down-regulated DEGs were mainly enriched in the pathways associated with cancer. *VEGFA* and *VEGFB* were found to be the initiating factor of VEGF signaling pathway. In addition, in the epidermal growth factor receptor (*EGFR*), *VEGFA* and *VEGFB* associated sub-network, kinase insert domain receptor (*KDR*), fibronectin 1 (*FN1*), transforming growth factor beta induced (*TGFBI*) and proliferating cell nuclear antigen (*PCNA*) were found to interact with at least two of the three hub genes. The DEGs in this sub-network were mainly enriched in Gene Ontology terms related to cell proliferation.

**Conclusion:**

*EGFR*, *KDR*, *FN1*, *TGFBI* and *PCNA* may interact with *VEGFA* to play important roles in NSCLC tumorigenesis. These genes and corresponding proteins may have the potential to be used as the targets for either diagnosis or treatment of patients with NSCLC.

## Introduction

Lung cancer ranks highest in both morbidity and mortality in most parts of the world [1, 2], and its absolute incidence is increasing dramatically [3]. Lung cancer can be categorized mainly into small cell and non-small cell histological subtypes. Among them, non-small cell lung cancer (NSCLC) is the most common form and accounts for almost 75% to 80% of lung cancer [2, 4]. Currently, about 70% newly diagnosed patients with either subtype of lung cancer suffer from local recurrence or metastatic lesions after resection, resulting in poor long-term survival rate [5]. Therefore, it is important to elucidate the mechanisms of lung cancer progression for the effective treatment of the disease.

Various studies have demonstrated that angiogenesis is essential for NSCLC growth and metastasis [6–8]. Vascular endothelial growth factor (VEGF), an angiogenic specific stimulator, has been found to regulate the growth of neoplastic angiogenesis and plays an important role in vascularization in different types of cancers [9, 10]. Bergers and Benjamin found that VEGF were highly expressed in the tumor microenvironment and strongly induced tumor angiogenesis [11]. Zhao *et al*. [12] suggested that angiogenesis and VEGF expression in lung cancer cells was attributed to increased activity of JAK2/STAT3 signal pathway which was associated with a decreased survival rate in cancers. In addition, Chen *et al*. [13] reported that the over-expression of VEGF was a poor prognostic factor in small-cell lung cancer. Therefore, strategies to inhibit tumor-associated angiogenesis may be promising in limiting NSCLC progression.

Despite progresses achieved in the management of NSCLC, identification of specific molecular criteria is still a challenge to increase response rates [14]. In the present study, we analyzed the gene expression profiles (GSE39345) of peripheral blood mononuclear cells (PBMC) in patients with advanced stage NSCLC. The differentially expressed genes (DEGs) between healthy controls and patients before chemotherapy were analyzed. To the best of our knowledge, the dataset has not been analyzed before. Particularly, we evaluated VEGF genes expression and analyzed the VEGF signaling pathway. The *VEGFA* sub-network was constructed and functional enrichment analysis were performed with its related DEGs. We aimed to identify the expression pattern of *VEGF* in NSCLC and to explore its potential correlation with the progression of NSCLC. Unlike previous studies that explored the function of *VEGF* through experimental methods, the bioinformatics analyses performed in study provided a comprehensive evaluation of VEGF related protein-protein interactions and could be used to predict the interaction relationships between *VEGF* and other genes.

## Materials and Methods

### Affymetrix Microarray Data

The microarray data GSE39345 used in our study was downloaded from the Gene Expression Omnibus (http://www.ncbi.nlm.nih.gov/geo/) database. This dataset analyzed the gene expression profiles of PBMC in patients with advanced stage NSCLC based on the platform of Illumina humanRef-8 v2.0 expression beadchip (GPL1604) (Affymetrix Inc., Santa Clara, California, USA). The gene expression profiles consisted of 20 healthy controls (HC), 32 patients before chemotherapy and 17 patients after chemotherapy. In this study, the datasets from 20 HC and 32 NSCLC samples before chemotherapy were analyzed. The study was approved by the Institutional Review Board of Kaohsiung Chang Gung Memorial Hospital, Taiwan. Samples were collected after informed consent had been obtained from the patients. The patient records were de-identified prior to analysis [15].

### Data Preprocessing and Differential Expression Analysis

The original array data were performed background correction and quartile data normalization. Then the DEGs between HC and NSCLC samples were identified based on the R/Bioconductor package limma [16]. The absolute value of log_2_-fold change (log_2_FC) ≥ 1.5 and p-value < 0.05 were considered as cutoff value.

### Pathway Enrichment Analysis

Kyoto Encyclopedia of Genes and Genomes (KEGG, http://www.genome.jp/) [17] is a collection of online database composed by the known genes and their biochemical functionalities. The Database for Annotation, Visualization and Integrated Discovery (DAVID, http://david.abcc.ncifcrf.gov/) [18] is a comprehensive set of functional annotation tool for relating the functional terms with gene lists by clustering algorithm. In order to analyze the DEGs in functional level, KEGG pathway enrichment analysis was performed using the DAVID online tool. The p-value < 0.05 was set as the threshold value.

### *VEGF* Genes and VEGF Signaling Pathway

VEGF family members play important roles in the progression of NSCLC. In the present study, the distribution of DEGs in VEGF signaling pathway was studied using the KEGGParser [19] plugin for cytoscape (www.cytoscape.org) [20].

### Protein-protein Interaction Network Construction

We downloaded the comprehensive interaction information of human proteins from the Search Tool for the Retrieval of Interacting Genes (STRING) database (http://string-db.org/) [21]. Then the interaction relationships of NSCLCL DEGs were extracted to construct the protein-protein interaction (PPI) network (combined score > 0.4) using cytoscape.

### Sub-network Construction

Study has found that the VEGFR and EGFR pathways are linked in solid tumors, particularly with respect to angiogenesis [22]. In our study, we extracted *EGFR*, *VEGFA* and *VEGFB*, and their interactions with the other DEGs from the PPI network. Then the sub-network associated with *EGFR*, and *VEGF* was constructed. In addition, the DEGs in the sub-network were performed Gene Ontology (GO) enrichment analysis using the BiNGO [23] plugin for cytoscape (p-value < 0.05).

## Results

### Identification of DEGs

In total, 3028 DEGs were obtained from both NSCLCL and HC samples. Thereinto, 1666 were up-regulated and 1542 were down-regulated.

### Pathway Enrichment Analysis

The result of pathway enrichment showed that the up-regulated DEGs were mainly enriched in 11 pathways, such as olfactory transduction, metabolism of xenobiotics by cytochrome P450 and drug metabolism. The down-regulated DEGs were mainly enriched in the pathways associated with cancer, such as Wnt signaling pathway and Notch signaling pathway.

### *VEGF* genes and VEGF Signaling Pathway

**Fig 1:**
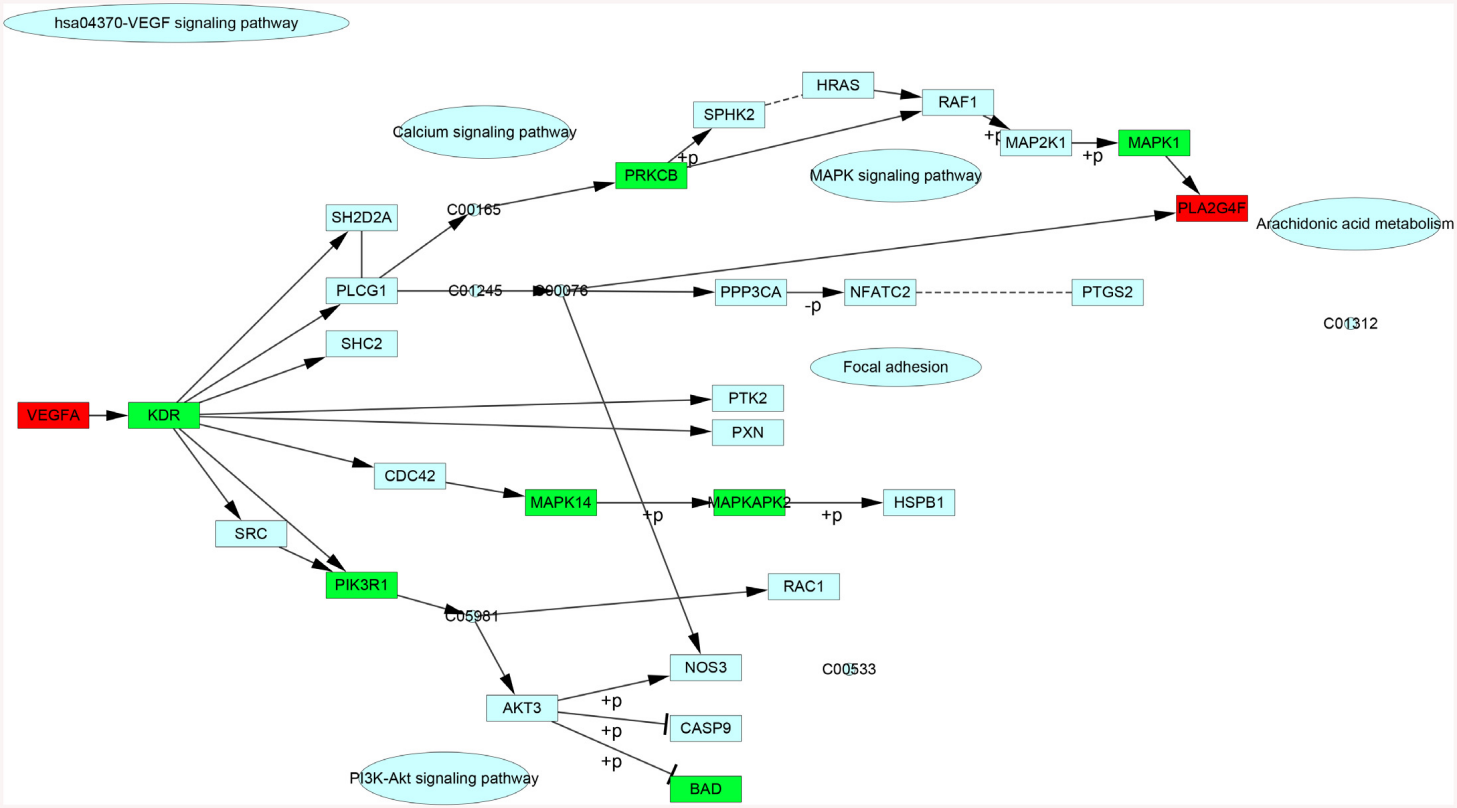
The differentially expressed genes (DEGs) distribution in vascular endothelial growth factor (VEGF) signaling pathway. Red color represents up-regulated DEGs and green represents down-regulated DEGs.

Among the DEGs, *VEGFA* and *VEGFB* were found to be the initiating factors of VEGF signaling pathway. After analysis of the distribution of DEGs in VEGF signaling pathway, down-regulated DEG of kinase insert domain receptor (*KDR*) was found to be a bottle neck factor of the pathway (Fig 1).

### PPI Network Analysis

The constructed PPI network contained 113797 interaction pairs between 2511 DEGs and the PPI networks obeyed the scale-free attribution (correlation = 0.859, R-squared = 0.888). The top 10 hub genes were shown in Table 1, including jun proto-oncogene, proliferating cell nuclear antigen (*PCNA*), epidermal growth factor receptor (*EGFR*) and *VEGFA*.

**Table 1:** The top 10 hub genes in the protein-protein interaction network.

Node	DE_state	Degree
JUN	-1	140
PCNA	-1	134
EGFR	1	128
CD4	1	127
VEGFA	1	122
IL6	-1	117
MAPK1	-1	117
CTNNB1	-1	115
GAPDH	1	109
PIK3R1	-1	107

DE_state represents differential expression state; 1 represents up-regulated differentially expressed genes (DEGs); -1 represents down-regulated DEGs.

### Sub-network Construction

**Fig 2:**
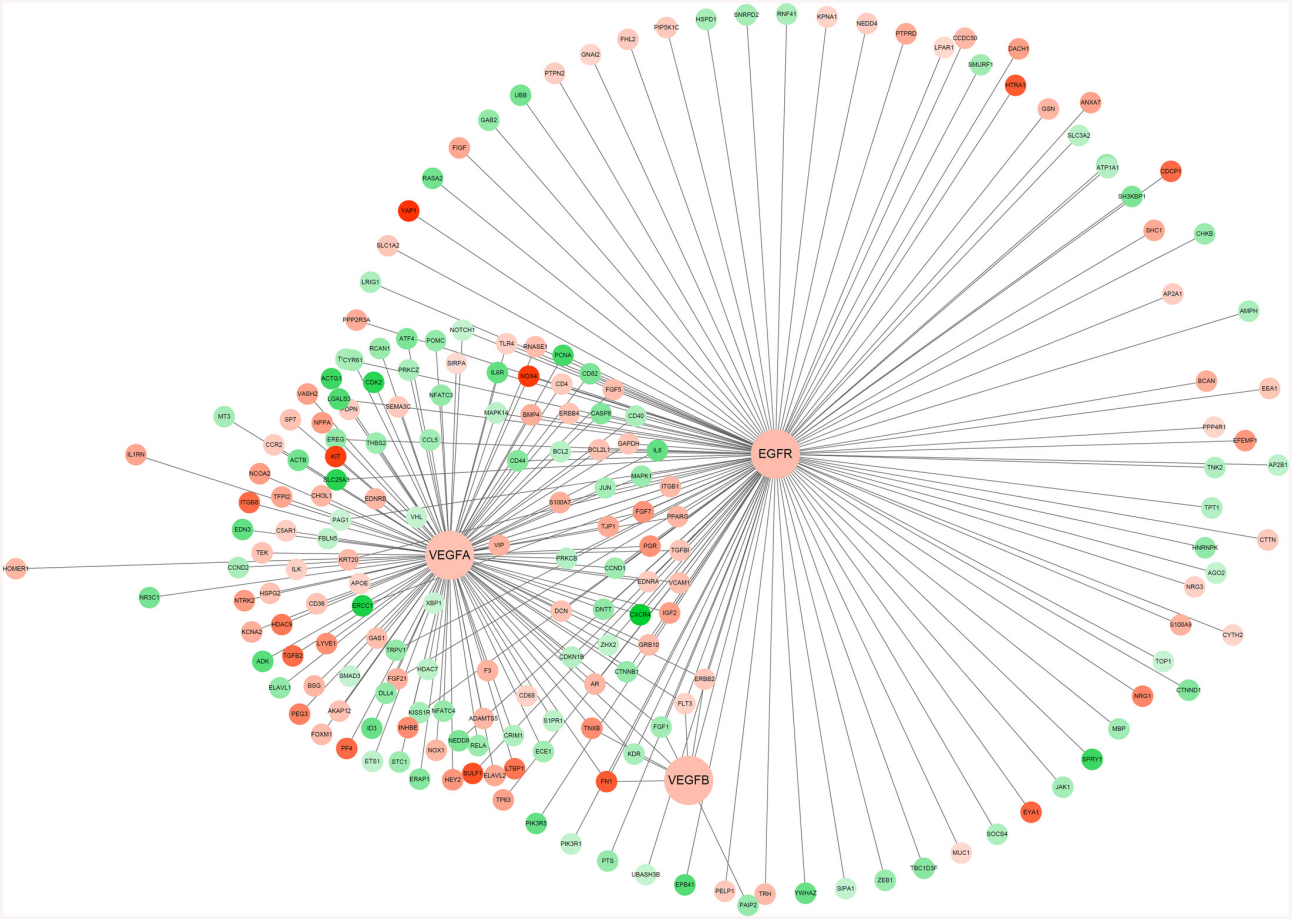
The vascular endothelial growth factor (*VEGF*) and epidermal growth factor receptor (*EGFR*) associated sub-network. Red color represents up-regulated differentially expressed genes (DEGs) and green represents down-regulated DEGs.

The *EGFR* and *VEGF* associated sub-network was shown in Fig 2. In the sub-network, *KDR* and fibronectin 1 (*FN1*) interact with all of the three genes. Transforming growth factor beta (*TGFBI*) and *PCNA* interact with *EGFR* and *VEGFA*. Additionally, the DEGs in this sub-network were mainly enriched in the GO biological process terms related to cell proliferation (Table 2).

**Table 2:** The top 10 significant Gene Ontology (GO) biological process terms enriched by the differentially expressed genes in sub-network.

GO-ID	Corrected p-value	Description
42127	7.95E-28	regulation of cell proliferation
8284	4.60E-27	positive regulation of cell proliferation
48522	6.95E-27	positive regulation of cellular process
48518	2.83E-25	positive regulation of biological process
42221	9.97E-23	response to chemical stimulus
48513	8.15E-22	organ development
51272	2.15E-21	positive regulation of cellular component movement
48856	2.15E-21	anatomical structure development
48731	2.15E-21	system development
50793	9.28E-21	regulation of developmental process

## Discussion

Highly treatment-resistant NSCLC is one of the major causes of cancer death all over the world and angiogenesis has become an integral process in promoting the growth and metastasis of NSCLC. As a result, therapy against VEGF, one of the major mediators of angiogenesis, may be an critical approach for regressing NSCLC [6]. In this study, *VEGFA* and *VEGFB* were found to be the initiating factors of VEGF signaling pathway. In addition, *KDR* was found to be a bottle neck factor of this pathway. Moreover, in the *EGFR* and *VEGF* associated sub-network, *KDR*, *FN1*, *TGFBI* and *PCNA* were found to interact with at least two of the three hub genes.

VEGF gene belongs to the platelet derived growth factor/VEGF growth factor family. Its protein product promotes endothelial cell proliferation and migration, and prevents cell apoptosis. Importantly, it is also involved in tumor-associated angiogenesis [24]. In our study, *VEGFA* was found to be the initiating factor of VEGF signaling pathway. Besides, it was a hub gene in the PPI network. Deng *et al*. [25] has reported that *VEGFA* is implicated in the carcinogenesis of numerous cancers including lung cancer. The *VEGFA* expression has been suggested to be related to the relapse and poor prognosis of NSCLC [26]. Specially, in NSCLC, the tumor progression and metastasis are considered to be associated with the mutations of some tumor suppressor genes, such as *p53*, and the mutation of *p53* induces VEGF gene expression [27]. Interestingly, KDR, the receptor of VEGF, was found to be down-regulated in the VEGF signaling pathway, acting as a bottle-neck factor of this pathway. Thus, we speculate that KDR may negatively regulate VEGF signaling pathway. Taken together, *VEGFA* and *KDR* may be potentially involved in the progression of NSCLC.

Currently, many drugs that target either VEGFR or EGFR signaling pathways in advanced NSCLC have been clinically validated [28]. EGFR, a member of the epidermal growth factor family, plays diverse biological roles in promoting malignancy including regulation of cell survival or apoptosis, and cell motility or metastasis, which makes EGFR an attractive therapeutic target [29, 30]. De Luca *et al*. [31] reported that activation of EGFR by TGF-α can increase the production of VEGF in human cancer cells.

In this study, we also found that *EGFR* was also involved in the *VEGF* and *EGFR* associated sub-network, in addition to the GO terms related to cell proliferation. In this sub-network, *KDR*, *FN1*, *TGFBI* and *PCNA* were found to interact with 2 or 3 of the 3 hub genes. *FN1* encodes fibronectin, a glycoprotein in extracellular matrix (ECM), which has been implicated in the development of many types of cancer. In lung cancer, fibronectin can promote lung cancer cell migration by activating FAK signaling [32]. *TGFBI* is an extracellular matrix (ECM) protein induced by transforming growth factor-β (TGF-β) in human cancer cells [33]. *TGFBI* has been discovered as a gene induced in the lung cancer cell line A549 by TGF-β [34]. Park *et al*. [35] has reported that *TGFBI* stimulates adhesion, proliferation and migration of renal proximal tubular epithelial cells. TGFBI can sustain cell adhesion to substrates, which may increase cell motility and metastases [36]. In addition, TGFBI-derived proteolytic peptides are pro-apoptotic in several cell types, which may be due to the activation of cell-specific proteases capable of degrading TGFBI [37]. Therefore, *TGFBI* may be used as a potential target for NSCLC treatment.

Furthermore, the down-regulated DEG of *PCNA* was identified in the PPI network and sub-network. In normal cells, the proliferation and apoptosis are under the control of cell cycle regulatory systems. The uncontrolled cell proliferation is considered to be the hallmark of cancer cells, thus, the proliferative potential of cancer cells may be an important prognostic factor [38]. Some antibodies including PCNA have been used to investigate the cell proliferation [39]. Zienolddiny *et al*. [40] has reported that PCNA is a marker for the evaluation of cell proliferative activity in lung cancer. Therefore, *PCNA* may participate in the regulation network of *VEGFA* to play an important role in NSCLC tumorigenesis and serve as a potential molecular marker associated with NSCLC.

In conclusion, our study provides a comprehensive bioinformatics analysis of the expression of VEGF in NSCLC. DEGs such as *VEGFA*, *VEGFB*, *EGFR*, *FN1*, *TGFBI*, *KDR* and *PCNA*. Their related GO terms may play important roles in NSCLC tumorigenesis and progression and have the potential to be used as targets for NSCLC diagnosis and treatment. Further genetic and experimental studies with larger sample size are needed to confirm our results.
